# Dynamics of Intraprocedural Dominant Frequency Identifies Ablation Outcome in Persistent Atrial Fibrillation

**DOI:** 10.3389/fphys.2021.731917

**Published:** 2021-10-12

**Authors:** Alain Pithon, Anna McCann, Andréa Buttu, Jean-Marc Vesin, Patrizio Pascale, Mathieu Le Bloa, Claudia Herrera, Chan-Il Park, Laurent Roten, Michael Kühne, Florian Spies, Sven Knecht, Christian Sticherling, Etienne Pruvot, Adrian Luca

**Affiliations:** ^1^Service of Cardiology, Lausanne University Hospital, University of Lausanne, Lausanne, Switzerland; ^2^Applied Signal Processing Group, Swiss Federal Institute of Technology, Lausanne, Switzerland; ^3^Department of Cardiology, Hôpital de La Tour, Geneva, Switzerland; ^4^Department of Cardiology, Inselspital, Bern University Hospital, University of Bern, Bern, Switzerland; ^5^Department of Cardiology, University Hospital of Basel, Basel, Switzerland

**Keywords:** atrial fibrillation, catheter ablation, dominant frequency, intracardiac electrogram, surface electrocardiogram, decision tree model

## Abstract

**Background:** The role of dominant frequency (DF) in tracking the efficiency of a stepwise catheter ablation (step-CA) in persistent atrial fibrillation (peAF) remains poorly studied. We hypothesized that the DF time-course during step-CA displays divergent patterns between patients in whom a step-CA successfully restores long-term sinus rhythm (SR) and those with recurrence.

**Methods:** This study involved 40 consecutive patients who underwent a step-CA for peAF (sustained duration 19 ± 11 months). Dominant frequency was computed on electrograms recorded from the right and left atrial appendages (RAA; LAA) and the coronary sinus before and during the step-CA synchronously to the 12-lead ECG. Dominant frequency was defined as the highest peak within the power spectrum.

**Results:** Persistent atrial fibrillation was terminated by a step-CA in 28 patients [left-terminated (LT)], whereas 12 patients remaining in AF after ablation [not left-terminated (NLT)] were cardioverted. Over a mean follow-up of 34 ± 14 months, all NLT patients had a recurrence. Among the 28 LT patients, 20 had a recurrence, while 8 remained in SR throughout follow-up. The RAA and V_1_ DF had the best predictive values of the procedural failure to terminate AF (area under the curve; AUC 0.84, *p* < 0.05). A decision tree model including a decrease in LAA DF ≥ 6.61% during the first 20 min following pulmonary vein isolation (PVI) and a baseline RAA DF <5.6 Hz predicted long-term SR restoration with a sensitivity of 83% and a specificity of 93% (*p* < 0.05).

**Conclusion:** This study found that high baseline DF values are predictive of unfavorable ablation outcomes. The reduction of the LAA DF at early ablation steps following PVI is associated with procedural AF termination and long-term SR maintenance.

## Introduction

Pulmonary vein isolation has emerged as an effective treatment for patients suffering from paroxysmal atrial fibrillation (AF), while its success rate in persistent AF (peAF) is significantly lower (Brooks et al., 2010). An extra-pulmonary vein (PV) substrate ablation, including the ablation of complex fractionated atrial electrograms (CFAEs) and/or the creation of linear lesions, may be beneficial (Nademanee et al., 2008; Brooks et al., 2010), but extensive ablation lesions are associated with increased procedural time and a high incidence of post-procedural atrial tachycardia.

It has been shown that the clinical efficacy of a stepwise catheter ablation (step-CA) is associated with a decrease in AF complexity as indicated by changes in the dominant frequency (DF) of AF (Lemola et al., 2006; Johner et al., 2020; Ma et al., 2021). However, whether the time-course of AF organization throughout an ablation may serve as an indicator of the efficacy of the procedure remains to be determined.

We recently showed that patients with peAF that are unresponsive to step-CA displayed higher AF complexity at baseline as indicated by higher DF values (Luca et al., 2020) and lower ECG-based organization indices (Buttu et al., 2016; McCann et al., 2021) than patients with successful ablation. Furthermore, we found that a step-CA led to increases in surface ECG AF organization in most patients (McCann et al., 2021), without significant differences between patients who remained in sinus rhythm (SR) in the long term and those who did not. In this study, we hypothesized that combining baseline intracardiac DF and its time-course throughout an ablation may be used to track the efficacy of step-CA *en route* to long-term SR restoration in peAF.

## Methods

### Patient Population and Ablation Procedure

This study has been performed within the framework of an ongoing project (REORGANIZE-AF) that is aimed at assessing the level of ECG and intracardiac electrogram (EGM) organization in peAF to improve the selection of patients for ablation. The study group consisted of 40 consecutive patients with peAF referred for a first step-CA. The patients suffered from AF for 6 ± 4 years, sustained for 19 ± 11 months before the ablation, and were resistant to pharmacological or electrical cardioversion. The details of the clinical characteristics of the study population are provided in Table 1.

**Table 1:** Clinical characteristics.

	**All** ***n* = 40**	**NLT** ***n* = 12**	**LT_Rec** ***n* = 20**	**LT_SR** ***n* = 8**	***p*-value^a^**	***p*-value^b^**	***p*-value^c^**
Age (yrs)	63 (56; 65)	63 (58; 64)	63 (53; 67)	60 (58; 63)	0.98	0.56	0.65
Sex (male/female)	38/2	12/0	18/2	8/0	0.26	-	0.35
AF duration (yrs)	6 (2; 8)	5 (2; 7)	5 (3; 6)	9 (6; 11)	0.74	0.03	0.07
Duration of sustained AF (mo)	15 (12; 24)	22 (14; 39)	13 (12; 24)	20 (10; 24)	0.05	0.28	0.60
BMI (kg/m^2^)	30 (25; 31)	30 (24; 31)	30 (25; 34)	29 (27; 30)	0.63	0.94	0.72
High blood pressure	27 (68)	8 (67)	13 (65)	6 (75)	0.72	0.69	0.68
Valvular disease	6 (15)	1 (8)	3 (15)	2 (25)	0.58	0.31	0.53
Diabetes	6 (15)	2 (17)	3 (15)	1 (13)	0.90	0.80	0.86
Tobacco	8 (20)	2 (17)	4 (20)	2 (25)	0.81	0.65	0.77
Hypercholesterolemia	18 (45)	6 (50)	9 (45)	3 (38)	0.78	0.58	0.72
Coronary artery disease	0 (0)	0 (0)	0 (0)	0 (0)	-	-	-
Sleep apnea syndrome	23 (58)	6 (50)	13 (65)	4 (50)	0.40	>0.99	0.46
Chronic kidney disease	1 (3)	0 (0)	1 (5)	0 (0)	0.43	-	0.52
CHA_2_DS_2_-Vasc score	1 (1; 2)	1 (0; 1)	2 (1; 2)	1 (1; 2)	0.12	0.41	0.47
Dilated cardiomyopathy	14 (35)	3 (25)	9 (45)	2 (25)	0.26	>0.99	0.33
Hypertrophic cardiomyopathy	3 (8)	0 (0)	2 (10)	1 (13)	0.26	0.21	0.85
Left ventricular fraction ejection (%)	50 (40; 60)	55 (50; 60)	50 (35; 56)	55 (44; 55)	0.18	0.50	0.47
Left atrial volume (*ml*)	168 (143; 193)	174 (160; 193)	162 (140; 190)	167 (139; 204)	0.41	0.64	0.90
**During stepwise catheter ablation**							
Cumulative ablation time (min)							
Total	56 (48; 68)	76 (61; 81)	55 (50; 60)	40 (29; 54)	<0.01	<0.01	0.04
PVI	22 (18; 26)	19 (16; 23)	25 (20; 28)	22 (16; 25)	0.11	0.76	0.39
CFAEs and linear ablation	33 (21; 49)	55 (48; 59)	26 (20; 40)	19 (5; 30)	<0.01	<0.01	0.09
Beta-blockers	28 (70)	7 (58)	15 (75)	6 (75)	0.33	0.44	>0.99
Calcium channel blockers	8 (20)	2 (17)	4 (16)	2 (25)	0.82	0.65	0.77
Amiodarone	7 (18)	1 (8)	4 (16)	2 (25)	0.38	0.31	0.77
Other antiarrhythmics	5 (13)	3 (25)	2 (10)	0 (0)	0.26	0.13	0.35
No. of antiarrhythmics drugs	2 (1; 3.5)	2 (2; 3)	3 (2; 3)	2 (2; 2)	0.03	0.27	0.43
Statins	5 (13)	3 (25)	2 (10)	0 (0)	0.26	0.13	0.35
**Recurrence type at follow-up**							
Persistent AF	4	3	1				
Paroxysmal AF	3	0	3				
ATs	25	9	16				
**Sites of AF termination**							
Roof	4		3	1			
Left atrial appendage	2		0	2			
Coronary sinus	4		3	1			
Mitral isthmus	8		6	2			
Pulmonary veins	2		0	2			
Septum	7		7	0			
Left lateral wall	1		1	0			

*Values are median with 25th and 75th percentiles or n (%)*.

a *NLT vs. LT_Rec*.

b *NLT vs. LT_SR*.

c *LT_Rec vs. LT_SR*.

*AF, atrial fibrillation; AT, atrial tachyarrhythmia; CFAEs, complex fractionated atrial electrograms; LT_Rec/LT_SR, left-terminated with/without recurrence at follow-up; NLT, not left-terminated; PVI, pulmonary veins isolation*.

All patients underwent a step-CA procedure consisting of pulmonary vein isolation (PVI), followed by left atrial (LA) CFAEs ablation and linear ablation (roof and mitral isthmus). The stepwise ablation protocol has been described previously (Buttu et al., 2016; Luca et al., 2020). The details of the ablation procedure are also provided in the Supplementary Material. The procedural endpoint was reached when AF terminated in SR or atrial tachycardia (AT). Patients with non-terminated peAF were electrically cardioverted. After the index ablation, all patients were followed and data were recorded at 3, 6, 12, 18, and 24 months, then every year. Recurrence was defined as AF or AT lasting more than 30 s (Calkins et al., 2017). All patients provided written informed consent, and the study was approved by the Human Research Ethics Committee of the Lausanne University Hospital.

Based on procedural and clinical outcomes, the study population was divided into three subgroups. Subgroup 1 (*n* = 8) consisted of patients in whom peAF was terminated into SR or AT by ablation and who remained arrhythmia free throughout the follow-up (left-terminated without recurrence—LT_SR). Subgroup 2 (*n* = 20) consisted of patients in whom peAF was terminated by ablation and who had a recurrence after the first step-CA procedure (left-terminated with recurrence—LT_Rec). Subgroup 3 (*n* = 12) consisted of patients in whom the step-CA procedure failed to terminate peAF (not left-terminated—NLT), all with recurrence during follow-up.

### Electrophysiological Study

More details are available in the Supplementary Material. The following catheters were introduced *via* the left and right femoral veins: a 3.5-mm cooled-tip catheter for mapping and ablation (Navistar Thermocool, Biosense Webster®, Irwindale, California), a circumferential duodecapolar Lasso® catheter (electrode spacing 2-6-2 mm, Biosense Webster®, Irwindale, California) within the LA, a quadripolar catheter (electrode spacing 5-5-5 mm, 4 mm electrode tip size, Supreme St Jude Medical®, Saint Paul, Minnesota) placed into the right atrial appendage (RAA), and a steerable decapolar catheter (electrode spacing 2-8-2 mm, 1 mm electrode tip size, Biosense Webster®, Irwindale, California) placed into the coronary sinus (CS), with the proximal electrode at the ostium. The ECG chest lead V_6_ was placed on the back (V_6b_) of the patients, within the cardiac silhouette, in order to better record LA activity (Luca et al., 2020). Furthermore, EGMs were synchronously recorded from the left atrial appendage (LAA), RAA, and CS at baseline, i.e., before the ablation, during PVI, and throughout CFAEs and the linear ablation. Surface ECG was continuously monitored at baseline and during the entire step-CA procedure. The ECG and EGM signals were recorded using an Axiom Sensis XP® System (Siemens®, Munich, Germany) at a sample rate of 2 kHz and bandpass filter settings of 0.5–200 and 30–400 Hz, respectively.

### Data Processing

The ECG and EGM signals were retrospectively processed using MatLab (The Mathworks Inc., Natick, MA, USA).

The EGM signals were rectified and bandpass filtered at 1–20 Hz (Botteron and Smith, 1995; Ng and Goldberger, 2007). Frequency spectra were estimated using the fast Fourier transform, and DF was identified as the highest peak frequency between 3 and 15 Hz. The EGMs with a DF power (1-Hz band centered at the DF peak) lower than 20% of the total power in the 3- to 15-Hz band were reviewed to exclude spurious DF values (Sanders et al., 2005). The surface ECG signals were bandpass filtered (1–50 Hz) to remove baseline wander and power line interference. Surface ECG DF estimation was preceded by ventricular activity cancelation in order to ensure the reliability of the ECG analysis during AF. The single beat method, in which the QRS and T waves are treated separately, was used as originally described by our group (Lemay et al., 2007). Following QRST cancellation, the power spectrum of each atrial ECG signal was computed using Welch's method (2.5-s Hamming window, 50% overlap) and the DF was defined as the frequency of the highest peak between 3 and 15 Hz.

**Figure 1:**
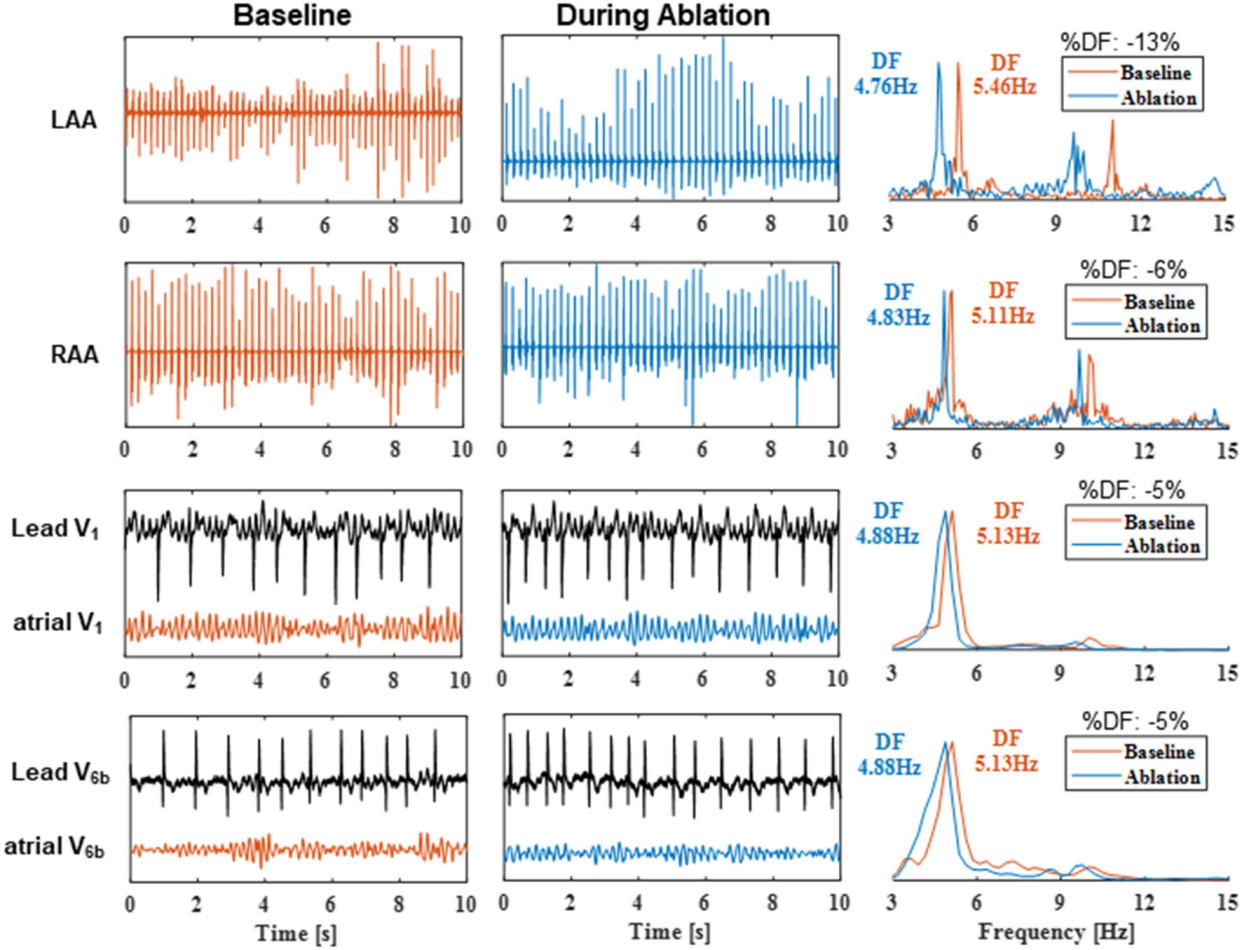
Dominant frequency estimation on 10-s epochs recorded from the LAA, RAA, and the ECG leads V_1_ and V_6b_ in an LT_SR patient at baseline and after 20 min of cumulative CFAEs and linear ablation. For the surface ECG recordings, the atrial V_1_ and V_6b_ signals represent the ECG leads V_1_ and V_6b_ devoid of ventricular activity. The corresponding power spectral densities (normalized by their maximum value) and the estimated DFs are illustrated in the right column. DF, dominant frequency; %DF, relative change in DF during ablation compared with baseline DF values. Other abbreviations as in previous tables.

The ECG and EGM signals were analyzed at different steps of the procedure: (i) baseline, (ii) during PVI, and (iii) during CFAEs and the linear ablation (LA ablation). The EGM DFs were computed on the distal dipole of the RAA catheter, on the Lasso® catheter placed within the LAA as the average DF of the 10 dipoles, and on the CS catheter as the average DF of the 5 dipoles. Because the atrial activity is best recognized in the leads V_1_ and V_6b_ (Luca et al., 2020), the ECG DFs were computed only on these two leads. The ECG and EGM signals were divided into 10-s epochs. Hence, for each 10-s epoch, a single DF value was available for each catheter (1 for the RAA distal dipole, 1 for the Lasso® catheter, and 1 for the CS catheter) and each ECG lead per patient. Interatrial EGM and ECG left-to-right DF gradients were obtained as the difference between LAA and RAA DFs and between V_6b_ and V_1_ DFs, respectively. Figure 1 shows an illustrative example of DF estimation on 10-s epochs simultaneously recorded from the LAA, RAA, and the ECG leads V_1_ and V_6b_ in an LT_SR patient at baseline and after 20 min of cumulative CFAEs and linear ablation. For each ECG recording, the atrial V_1_ and V_6b_ signals represent the ECG leads V_1_ and V_6b_ devoid of ventricular activity.

### Statistical Analysis

Continuous variables were expressed as the median and interquartile range (IQR) and categorical variables as numbers and percentages. The significance of any difference between subgroups was analyzed with the Mann–Whitney *U*-test for continuous variables and with the Fisher's exact test for categorical variables. A receiver-operator characteristic (ROC) analysis was performed to assess the performance of ECG and EGM DFs as predictors of ablation outcomes. The optimal ROC curve cutoff was defined as the combination of the highest sensitivity and specificity. A logistic regression analysis was used to determine the predictors of ablation outcomes and to compute the respective odds ratios (OR). Freedom from atrial arrhythmias >30 s during follow-up was analyzed using the Kaplan–Meier method, and a log-rank test was applied to compare differences between subgroups. A decision tree model based on baseline DF and its relative evolution during LA ablation were developed to intra-procedurally predict ablation outcomes. The statistical significance was set at *p* < 0.05. Analyses were performed using XLSTAT (ADDINSOFT®, Paris, France) or MatLab.

## Results

### Study Population

Persistent AF was terminated by ablation within the LA in 28 out of 40 patients (70%, LT group). Twelve patients (30%, NLT group) remained in AF at the end of the procedure and required electrical cardioversion to restore SR. The termination of AF occurred during PVI in 2 patients, during ablation of CFAEs in 14 patients, and after PVI plus CFAEs and linear ablation in 12 patients. After a single step-CA procedure, all 12 NLT patients developed a recurrence. Among the 28 LT patients, 20 had a recurrence (LT_Rec group), while 8 remained in SR throughout follow-up (LT_SR group). In the NLT and LT_Rec groups, recurrence occurred as AF (*n* = 7) and as ATs (*n* = 25) on average 7 ± 10 months after the index procedure. The baseline characteristics of the subgroups are presented in Table 1. A gradual and significant decrease in total ablation time was observed between the subgroups, with the longest time in NLT patients, intermediate in LT_Rec patients, and the shortest time in LT_SR patients (76 vs. 55 vs. 40 min, respectively; *p* < 0.05). Moreover, NLT patients had significantly longer ablation times during CFAEs and linear ablation than LT_Rec and LT_SR patients (55 vs. 26 vs. 19 min, respectively; *p* < 0.01). Clinical parameters and PVI ablation times were similar between subgroups. The mean follow-up duration for the study population was 34 ± 14 months, and at the end of the follow-up period, 34 (85%) patients were in SR without (28 out of 34, 82%) and with (6 out of 34, 18%) amiodarone, with a mean number of 2 ± 1 ablation procedures per patient.

### Baseline ECG and EGM DF Values

Table 2 shows the DF values computed at baseline (before ablation) on RAA, LAA, CS EGMs, and on ECG leads V_1_ and V_6b_ for the entire population and the three subgroups. Both ECG and EGM DFs were uniformly higher in NLT patients than in LT patients. Although there was no significant difference in DF between the LT_Rec and LT_SR patients, graded DF values were observed among the three subgroups, starting with the highest ones for NLT patients to the lowest ones for LT_SR patients. Particularly, NLT patients also displayed a negative LAA-to-RAA DF gradient [median (IQR): −0.32 (−0.58; 0.09) Hz], while that of LT patients was positive [LT_Rec: 0.19 (−0.04; 0.34) Hz; LT_SR: 0.28 (−0.23; 0.39) Hz]. In contrast, the surface V_6b_-to-V_1_ DF gradient was similar between the three subgroups. Altogether these results show that ECG and EGM DFs have the potential to refine the selection of patients with peAF unresponsive to ablation (NLT group).

**Table 2:** Surface ECG and intracardiac electrogram (EGM) DF values at baseline.

	**All** **(*n* = 40)**	**NLT** **(*n* = 12)**	**LT_Rec** **(*n* = 20)**	**LT_SR** **(*n* = 8)**	***p*-value^a^**	***p*-value^b^**	***p*-value^c^**
RAA	5.74 (5.32; 6.42)	6.69 (6.13; 7.12)	5.62 (5.25; 6.08)	5.37 (5.14; 5.80)	<0.001	<0.005	0.51
LAA	5.74 (5.55; 6.50)	6.26 (5.70; 6.79)	5.69 (5.42; 6.52)	5.57 (5.42; 5.91)	0.08	<0.05	0.5
CS	5.47 (4.88; 6.00)	5.73 (5.61; 6.07)	5.12 (4.83; 5.87)	5.26 (4.86; 5.69)	0.09	0.10	0.85
V_1_	5.78 (5.13; 6.35)	6.23 (5.98; 6.77)	5.49 (5.10; 5.92)	5.31 (5.13; 5.83)	<0.005	<0.01	0.95
V_6b_	5.49 (5.13; 5.86)	5.86 (5.55; 6.41)	5.19 (4.88; 5.62)	5.37 (5.13; 5.86)	<0.01	0.06	0.51
LAA-to-RAA DF gradient	0.14 (−0.32; 0.34)	–0.32 (−0.58; 0.09)	0.19 (−0.04; 0.34)	0.28 (−0.23; 0.39)	<0.05	0.11	0.99
V_6b_-to-V_1_ DF gradient	0.00 (−0.49; 0.06)	0.00 (−0.67; 0.00)	0.00 (−0.37; 0.03)	0.00 (−0.43; 0.24)	0.78	0.42	0.48

*Values are median (25th and 75th percentiles) Hz*.

a *NLT vs. LT_Rec*.

b *NLT vs. LT_SR*.

c *LT_Rec vs. LT_SR*.

*CS, coronary sinus; DF, dominant frequency; LAA, left atrial appendage; RAA, right atrial appendage; other abbreviations as in Table 1*.

### Baseline DF as a Predictor of Ablation Outcomes

**Figure 2:**
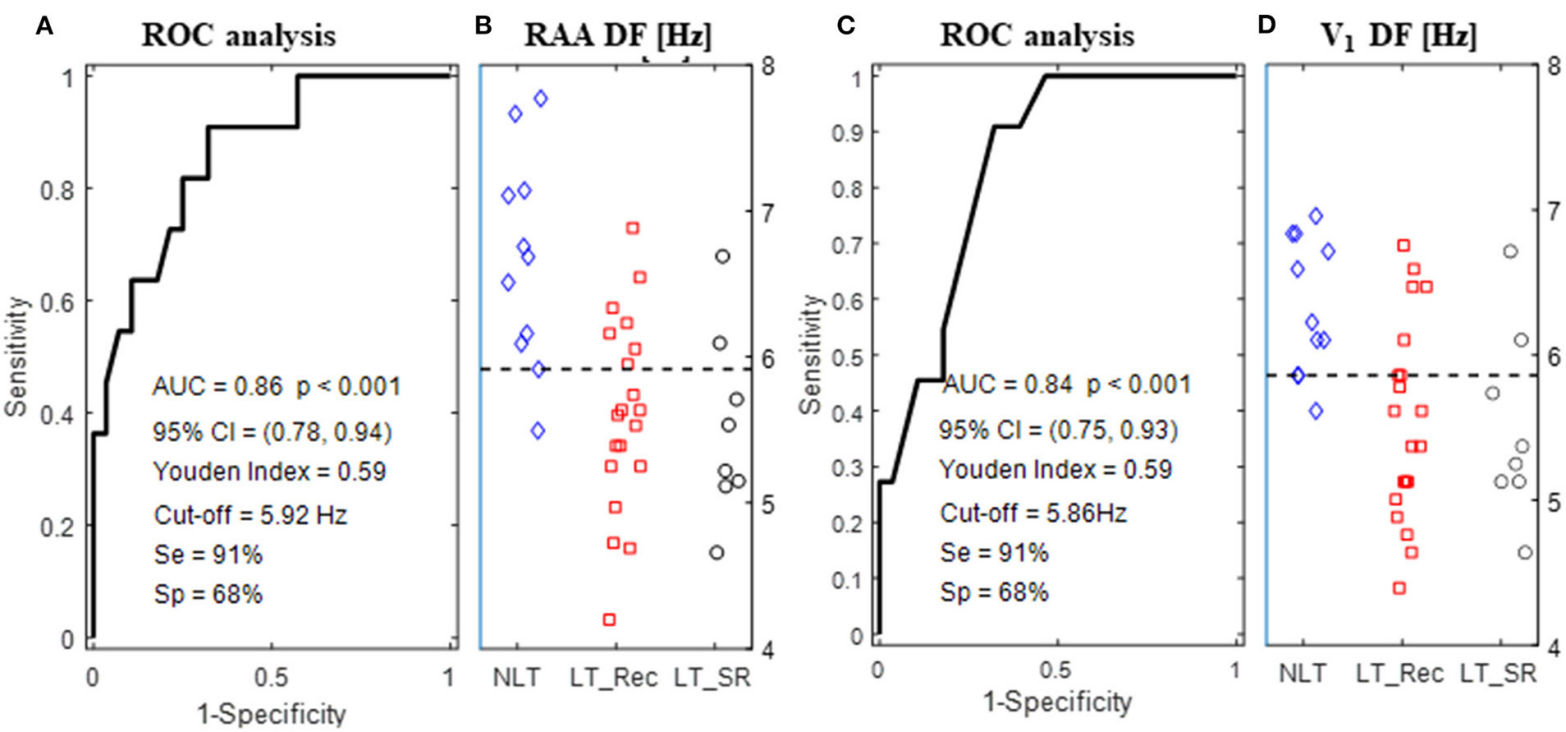
ROC curve for baseline DF computed from the RAA **(A)** and lead V_1_ **(C)**. **(B,D)** Show the distribution of RAA and V_1_ DF values, respectively, grouped by the procedural outcome (NLT, LT_Rec, and LT_SR). The horizontal dashed lines indicate the optimal cutoff points for DF above which both procedural AF termination and SR maintenance at follow-up were less likely to be achieved by ablation. Abbreviations as in Table 3.

Table 3 reports the predictive performances of baseline ECG and EGM DFs for the pre-ablation selection of NLT patients. A univariate logistic regression analysis showed that an increased DF in the RAA (OR 10.9), in LAA (OR 3.7), and on lead V_1_ (OR 8.5) and lead V_6b_ (OR 7.8), and a negative LAA-to-RAA DF gradient (OR 0.8) were all significantly (*p* < 0.05) associated with an unfavorable procedural outcome. The ROC curve analysis showed that an RAA DF ≥ 5.92 Hz (area under the curve; AUC = 0.86; Figure 2A) or a V_1_ DF ≥ 5.86 Hz (AUC = 0.84; Figure 2C) predicted the procedural outcome (*p* < 0.05) with a sensitivity of 91%, a specificity of 68%, and a PPV and NPV of 53 and 95%, respectively. Figures 2B,D show the distribution of DF values from the RAA and lead V_1_, respectively, grouped by procedural and clinical outcomes. Supplementary Table 1 shows the predictive performance of baseline ECG and EGM DFs for the pre-ablation identification of patients with long-term SR maintenance after a single CA procedure (LT_SR subgroup). No significant association was found between baseline DF and the restoration of long-term SR. In summary, high surface and intracardiac DF values and a negative LAA-to-RAA DF gradient before ablation are associated with the procedural failure to terminate AF.

**Table 3:** Baseline ECG and EGM DFs as predictors of procedural ablation outcomes (NLT patients vs. LT_Rec + LT_SR patients).

	**Odds ratio**			**ROC analysis**					
	**OR**	**95% CI**	***p*-value**	**AUC (95% CI)**	**Optimal cutoff**	**Se**	**Sp**	**PPV**	**NPV**
RAA	10.9	2.3–53.6	0.003	86% (0.78–0.94)	≥5.92 Hz	91%	68%	53%	95%
LAA	3.7	1.2–11.6	0.025	72% (0.57–0.88)	≥5.64 Hz	91%	50%	42%	93%
CS	2.65	0.89–7.92	0.07	70% (0.58–0.82)	≥5.61 Hz	82%	68%	50%	90%
V_1_	8.5	1.9–37.3	0.005	84% (0.75–0.93)	≥5.86 Hz	91%	68%	53%	95%
V_6b_	7.8	1.7–35.6	0.008	80% (0.67–0.93)	≥5.49 Hz	91%	61%	48%	94%
LAA-to-RAA DF gradient	0.80	0.66–0.97	0.02	74% (0.56–0.92)	≤-0.44 Hz	45%	93%	71%	81%

*AUC, area under the curve; CI, confidence interval; NPV, negative predictive value; OR, odds ratio; ROC, receiver operating characteristic; PPV, positive predictive value; Se, sensitivity; Sp, specificity; other abbreviations as in Table 2*.

### Effect of Ablation on DF

To assess the cumulative effect of ablation on AF organization, ECG and EGM DFs were computed during PVI and LA ablation (CFAEs and linear ablation) until AF termination or cardioversion. For each patient, DFs were first computed on 10-s epochs and then averaged over all available epochs at the end of PVI (end_PVI), during the first 10, 20, and 30 min of cumulative ablation following PVI, and the last 3 min of ablation (end_ABL). The relative change in DF was calculated as the percentage deviation of the average DF from the baseline DF value.

**Figure 3:**
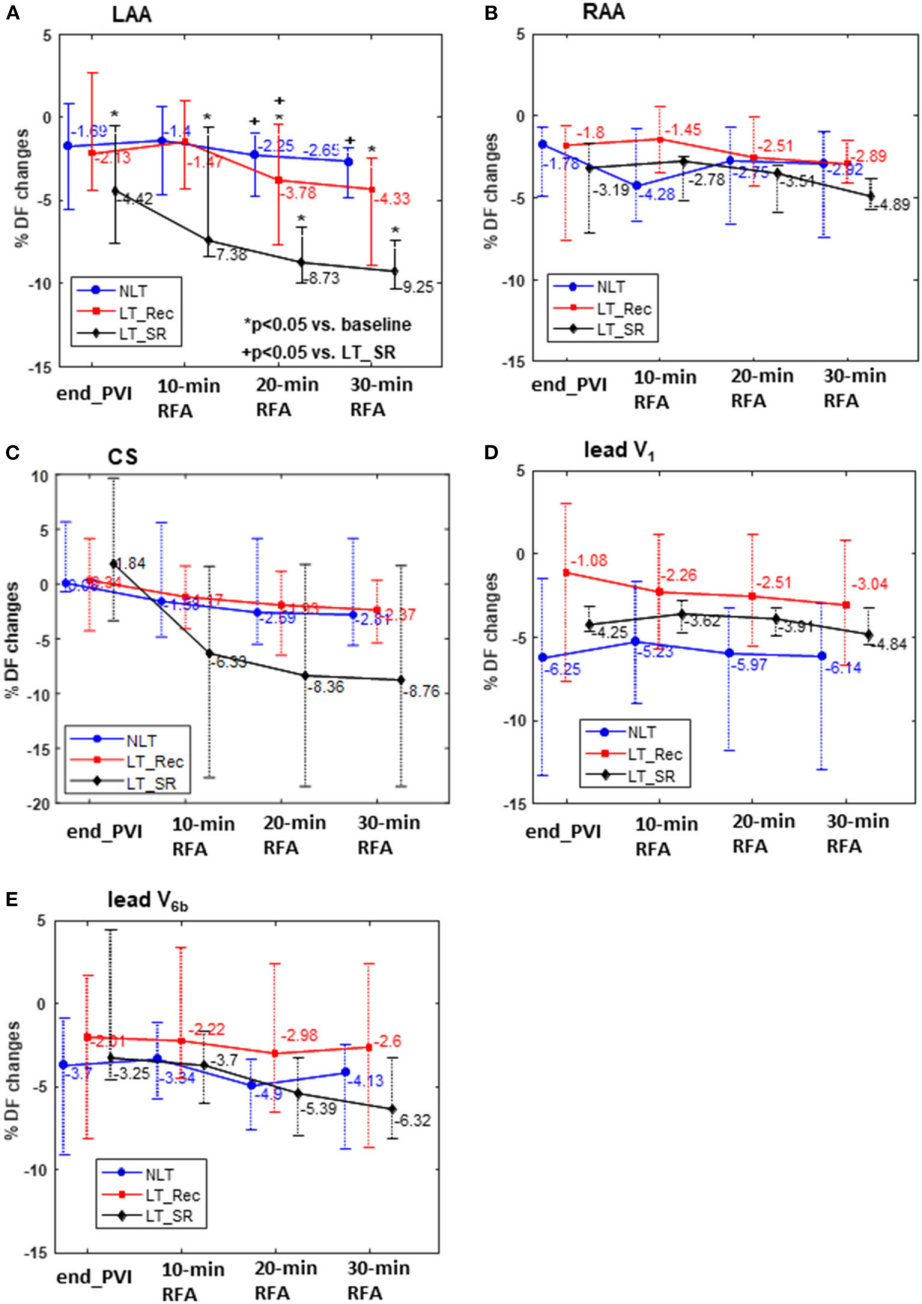
Relative changes (%) in DF compared with the baseline DF at the end of PVI (endPVI) and during the first 10, 20, and 30 min of cumulative ablation following PVI. DF was measured within the LAA, RAA, CS, and on the ECG leads V_1_ and V_6b_. RFA, radiofrequency ablation. Abbreviations as in previous tables and figures.

The only divergent patterns of DF values between the three subgroups during LA ablation were observed in the LAA. The temporal evolution of RAA, CS, V_1_, and V_6b_ DF was similar between subgroups (Figure 3). Supplementary Figure 2 shows that the subgroups displayed a similar decrease in DF at end_ABL compared with the baseline DF values. Supplementary Figure 3 shows that, for LT patients, the extra-PV substrate ablation led to the durable abolition of the baseline positive LAA-to-RAA DF gradient. In contrast, for NLT patients, after a transient phase of null LAA-to-RAA DF gradient during the first 30 min of LA ablation, the baseline negative LAA-to-RAA DF gradient was re-established at end_ABL.

**Figure 4:**
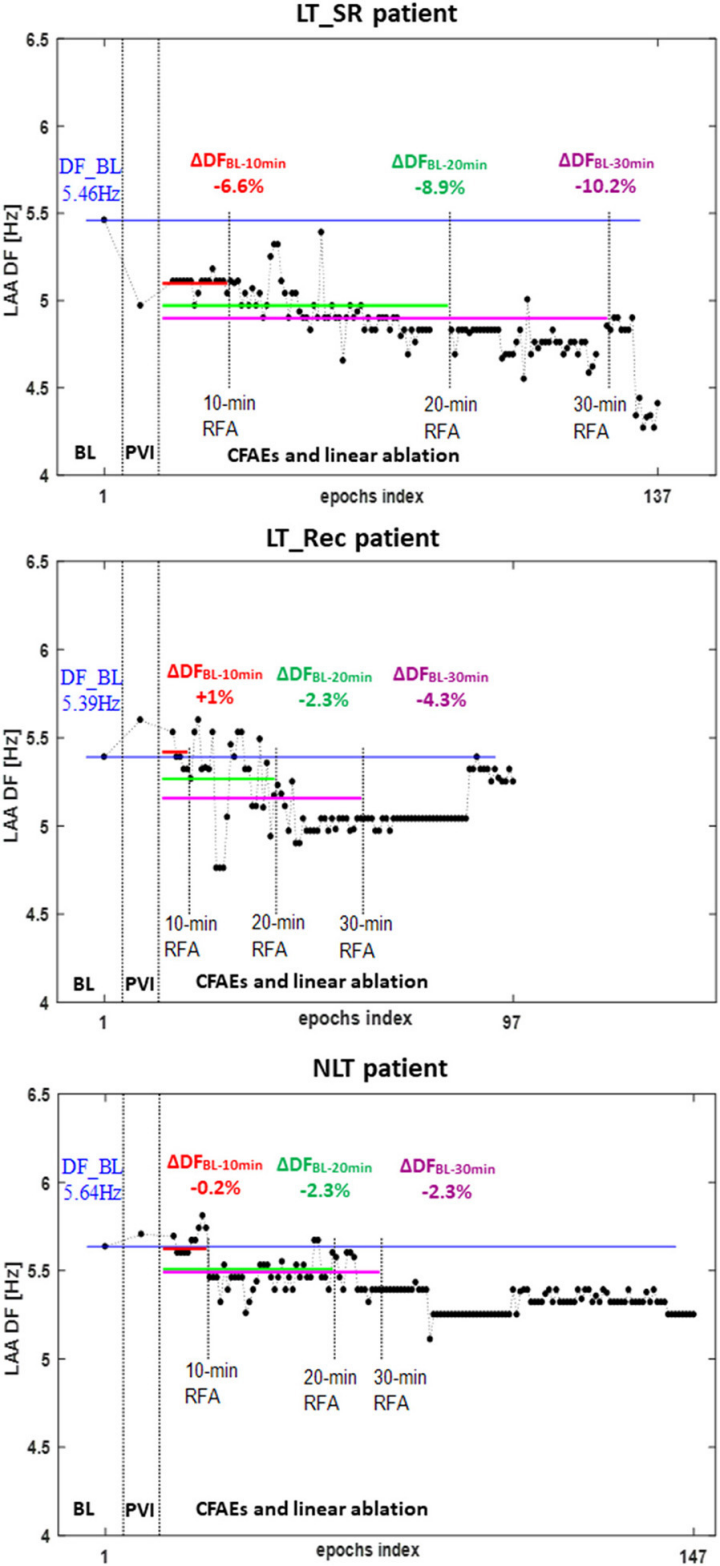
Temporal evolution of DF measured on 10-s epochs recorded from the LAA in an LT_SR patient (top), an LT_Rec patient (middle), and an NLT patient (bottom) at baseline (BL), at the end of PVI (end_PVI), and during LA ablation (CFAEs and linear ablation). Each dot represents the DF value computed on a single 10-s epoch. Average DFs at baseline and during the first 10, 20, and 30 min of cumulative ablation following PVI are represented by the horizontal blue, red, green, and magenta lines, respectively. PVI, pulmonary vein isolation; RFA, radiofrequency ablation; ΔDF, relative change in DF during ablation compared with the baseline DF value. Other abbreviations as in previous tables and figures.

Figure 4 shows an illustrative example of the temporal evolution of DF estimated on 10-s epochs acquired from the LAA at baseline, during PVI, and during LA ablation in an LT_SR, an LT_Rec, and an NLT patient. The average DF at baseline and during the first 10, 20, and 30 min of cumulative ablation within LA are indicated by horizontal blue, red, green, and magenta lines, respectively. For the LT_SR patient, the ablation led to the progressive reduction of the LAA DF from end_PVI throughout the LA ablation (ΔDF_BL−10min_ −6.6%, ΔDF_BL−20min_ −8.9%, ΔDF_BL−30min_ −10.2%). For the LT_Rec patient, the reduction in LAA DF only occurred after 20 min of CFAE ablation (ΔDF_BL−10min_ +1%, ΔDF_BL−20min_ −2.3%, ΔDF_BL−30min_ −4.3%), while for the NLT patient, no significant changes in DF occurred during the first 30 min of ablation. These specific findings were confirmed for the three subgroups (Figure 3A). The subgroup comparison shows that the relative changes in LAA DF at end_PVI and after the first 10 min of LA ablation were not significantly different. However, the LT_SR subgroup displayed significantly higher reduction of DF than the other two subgroups after 20 min of ablation within LA (LT_SR vs. LT_Rec vs. NLT: −8.73 vs. −3.78 vs. −2.25%, *p* < 0.05; Figure 3A). Altogether, these results suggested that tracking intracardiac DF in the LAA during complex ablation procedures may help identify patients without recurrence at follow-up.

### Changes in DF During Ablation as a Predictor of Clinical Outcome

The ROC analysis of the relative change in LAA DF after 20 min of LA ablation (time point of significant difference between subgroups) showed that a decrease in LAA DF ≥ 6.61% predicted long-term maintenance of SR, with 83% sensitivity, 74% specificity, 38% PPV, and 96% NPV (AUC = 0.75, 95% CI 0.64–0.86, *p* < 0.05; Supplementary Table 2; Supplementary Figure 4A). Supplementary Figure 4B shows that patients with an LAA DF decrease of ≥6.61% displayed a trend toward a lower recurrence rate than those with a decrease of <6.61% (40 vs. 5%; *p* = 0.055). Relative changes in RAA, CS, V_1_, and V_6b_ DF after 20 min of cumulative ablation within LA were not associated with the long-term maintenance of SR (Supplementary Table 2).

**Figure 5:**
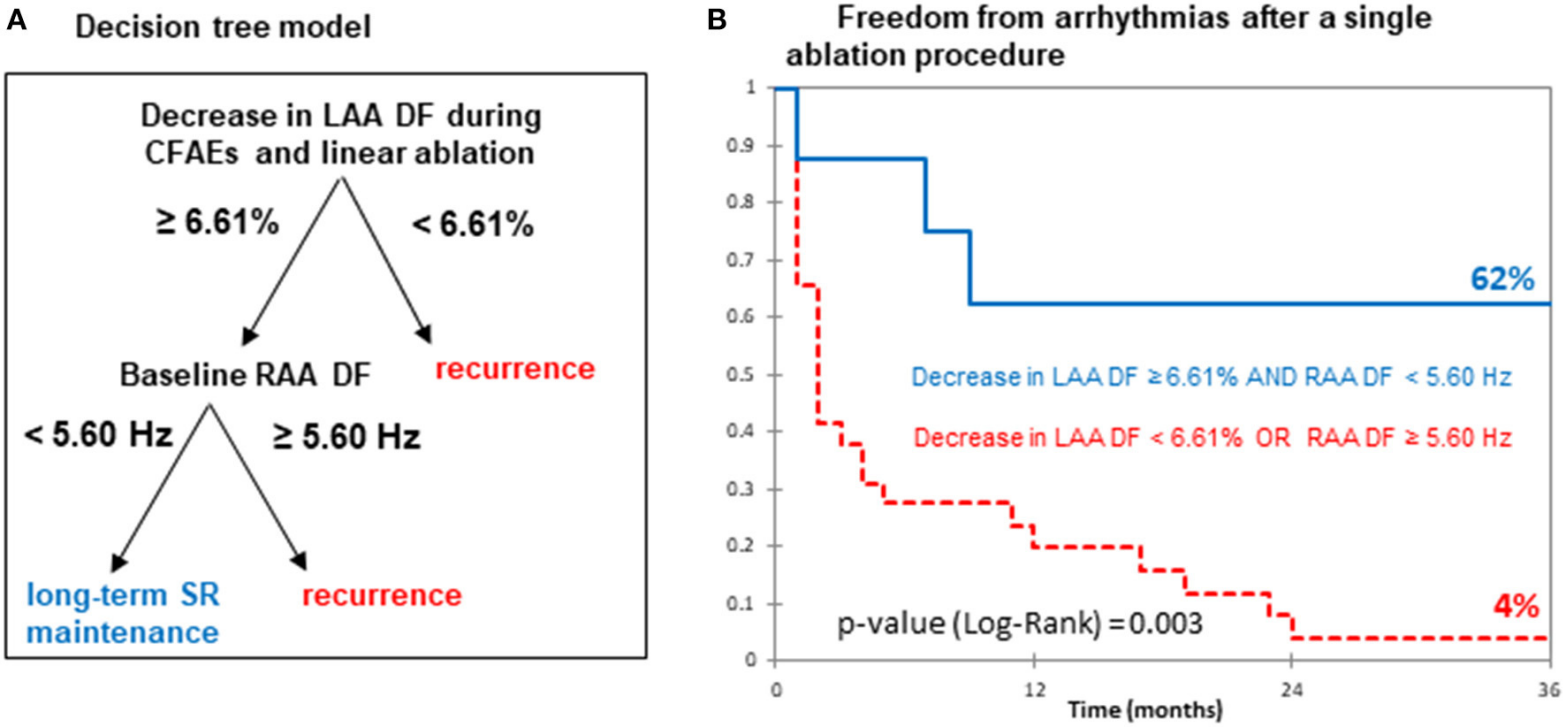
Predictive performance of DF for long-term SR maintenance. **(A)** Decision tree model based on the relative changes in LAA DF after the first 20 min of LA ablation and baseline RAA DF. **(B)** Kaplan–Meier curves for freedom from AF recurrence after catheter ablation. Abbreviations as in previous figures.

A decision tree model combining the relative change in LAA DF during ablation within LA and baseline RAA DF was developed to improve the prediction performances for long-term ablation outcomes (Figure 5A). This model was based on two steps: (1) a decrease of <6.61% in LAA DF after a 20-min ablation was associated with recurrence; (2) a baseline RAA DF <5.6 Hz in patients displaying an LAA DF decrease of ≥6.61% identified cases with the lowest risk of recurrence as shown by the Kaplan–Meier analysis (AF-free rate: 62 vs. 4%, *p* < 0.01; Figure 5B), with a sensitivity of 83%, a specificity of 93%, and a PPV and an NPV of 63 and 97%, respectively.

In summary, progressive LAA organization during ablation and low baseline RAA DF values are associated with the long-term maintenance of SR. These findings suggest that tracking the LAA DF during ablation may help define procedural ablation endpoints.

## Discussion

### Main Findings

This study presented new information regarding the clinical role of DF in predicting ablation outcomes and tracking of the efficacy of complex ablation procedures. First, the study confirmed that high surface and intracardiac DFs and a negative LAA-to-RAA DF gradient before ablation are associated with the procedural failure to terminate AF and high recurrence rates at follow-up. Second, it demonstrated that patients with a significant decrease in LAA DF during ablation and low RAA DF at baseline were more likely to remain in SR after a single ablation procedure. Altogether, these findings suggested that monitoring the intra-procedural evolution of DF may help assess the amount of ablation required to restore long-term SR in patients with long-standing peAF.

### High Baseline DF and Negative LAA-to-RAA DF Gradient Are Predictive of Unfavorable Procedural Ablation Outcomes

Extensive atrial remodeling is associated with suboptimal outcomes of catheter ablation in peAF (Nademanee et al., 2008; Brooks et al., 2010). Intracardiac DF is an acceptable surrogate for the degree of atrial remodeling, with high DF values indicative of advanced remodeling (Lemola et al., 2006; Brooks et al., 2010). Our group has recently shown that patients with peAF unresponsive to stepwise ablation had advanced bi-atrial and CS remodeling as shown by high surface and intracardiac DFs (Luca et al., 2020). The clinical role of DF in predicting procedural ablation outcomes has been investigated by several groups. Yoshida et al. (2011) found that patients without AF termination after both PVI and CFAE ablation had higher DFs in the LAA and on lead V_1_ than patients with procedural AF termination. Lo et al. (2009) showed that low bi-atrial DFs were associated with acute AF termination. Our study confirms that high DFs in the RAA, LAA, and ECG leads V_1_ and V_6b_ were associated with the procedural failure to terminate AF. Another important finding is that the RAA DF had the highest predictive accuracy, which is in line with previous studies reporting that non-PV foci such as in the right atrium (RA) can maintain peAF (Narayan et al., 2012; Hasebe et al., 2016). Narayan et al. (2012) in the CONFIRM trial found that up to one-third of the identified AF rotors or drivers were located in the RA. Hasebe et al. (2016) showed that AF initiated by RA triggers had a baseline positive RA-to-LA DF gradient. In our study, patients without AF termination by ablation within the LA had higher RAA and LAA DFs, negative LAA-to-RAA DF gradients, and longer ablation times than those of patients with successful ablation. These results supported the hypothesis of a high number of bi-atrial AF drivers in patients in whom AF persists despite extensive LA ablation. In summary, our findings suggested that bi-atrial DF values before ablation may help predict the procedural outcome.

### Temporal Evolution of DF During Stepwise Ablation

Extensive ablation has been shown to affect the atrial fibrillatory activity. Yokokawa et al. (2010) showed that linear ablation upon PVI resulted in a significant decrease both in the prevalence of major spectral components and in the DF of lead V_1_ and the CS. Johner et al. (2020) recently showed that stepwise extra-PV AF substrate ablation significantly affects the CS and RA DF. Our study evaluated the effect of PVI followed by CFAEs and linear ablation on the DF measured from RAA, LAA, CS, and ECG leads V_1_ and V_6b_. Surface and intracardiac DFs significantly dropped from baseline to the end of ablation for all the subgroups of patients. Importantly, the time-course of relative changes in the DF during ablation was significantly different between subgroups only for the DF measured in the LAA. In particular, LT_SR patients displayed a progressive reduction in the LAA DF during LA ablation, while NLT patients showed a decrease in the LAA DF only toward the end of ablation. In contrast, LT_Rec patients displayed an intermediate pattern. A few possibilities may explain the high sensitivity of LAA DF to cumulative ablation in the LT_SR group. LT_SR patients had short CFAEs ablation time and positive LAA-to-RAA DF gradient, suggesting a limited number of critical AF drivers within the LA (Haissaguerre et al., 2014). In contrast, patients without AF termination (NLT group) underwent extensive LA ablation before the occurrence of DF changes, suggestive of multiple bi-atrial AF drivers. A failure to decrease the LAA DF at the early stages of ablation may reflect the insufficient elimination of LA AF drivers. Recently, Honarbakhsh et al. (2018) reported that the ablation of AF drivers (rotational and focal) corresponding to sites of high atrial organization was more likely to cause LAA cycle length prolongation or AF termination. Altogether, these findings suggested that the intra-procedural evolution of LAA DF may be a useful marker of ablation efficacy *en route* to restoring long-term SR in peAF.

### Intracardiac DF and Long-Term Ablation Outcome

The present study showed that a ≥6.61% decrease in the LAA DF after 20 min of CFAE ablation was associated with long-term SR after a single ablation procedure. We found that a decision tree model combining the level of LAA DF decreases and baseline RAA DF values improves the specificity of SR maintenance. Among the patients with a decrease in LAA DF ≥6.61%, only those with baseline RAA DF <5.6 Hz had the lowest risk of arrhythmia recurrence. While the decrease in LAA DF may reflect the efficient elimination of LA AF drivers, a low baseline RAA DF value fits with mild RA remodeling, while high baseline RAA DF suggests multiple RA drivers (Narayan et al., 2012). In summary, a decision tree model combining the changes in LAA DF during ablation and baseline RAA DF values appears promising in guiding complex ablation procedures for restoring long-term SR in peAF.

### Clinical Implications

A prior study found that a ≥11% decrease in DF of lead V_1_ after PVI and CFAE ablation was as predictive of freedom from recurrences as AF termination (Yoshida et al., 2010). Acute AF termination may reflect the elimination of AF critical drivers (Oral et al., 2008; Honarbakhsh et al., 2018) and has long been thought of as an optimal ablation endpoint in peAF. Studies reporting the association of AF termination with long-term clinical success have shown conflicting results. While in the study of Scherr et al. (2015), procedural AF termination improved long-term outcomes in patients undergoing substrate-based ablation, in a substudy of the STAR AF II trial, acute termination did not predict long-term AF freedom (Kochhäuser et al., 2017). In our study, 70% of patients with AF termination developed a recurrence. Importantly, these patients did not display any significant decrease in LAA DF during the first 20 min of CFAE ablation, which is suggestive of our inability to eliminate critical AF drivers (Lemola et al., 2006). Moreover, these patients had longer ablation times than those without AF recurrence, suggesting that extensive LA substrate modification does not improve ablation success despite acute AF termination. These findings add to the bulk of studies showing the lack of long-term clinical benefit of stepwise ablation (Verma et al., 2015; Kochhäuser et al., 2017). In a previous study, Atienza et al. (2009) found in a mixed paroxysmal and persistent AF population undergoing ablation of DFmax sites and PVI that only those patients showing significant reductions in both LA and RA DFs with the abolition of the baseline LA-to-RA DF gradient remained free from AF. In our study, among patients displaying both baseline positive LAA-to-RAA DF gradient and its abolition by extra-PV substrate ablation, only those with significant reductions of the LAA DF at the early steps of ablation had the lowest risk of arrhythmia recurrence (LT_SR group). In conclusion, significant LAA DF reductions during ablation may be used as an indicator of the elimination of the AF drivers and ablation efficacy, but further studies are needed to validate this parameter in real-time settings.

### Limitations

First, this study is limited by the small size of the population, which might have resulted in overestimated performances of the predictive models. However, the patients were consecutively included, and the analysis was performed offline, preventing any selection bias. Second, the tracking of AF organization was performed on EGMs from a limited number of atrial sites (LAA, RAA, and CS). Because of the stepwise ablation at multiple LA sites, the LAA was intently chosen as the site for the tracking of AF organization as no ablation was applied in this structure. It is possible that other atrial sites would have identified different intraprocedural dynamics and threshold values for DF. Third, patients in whom AF was terminated by a step-CA also had shorter ablation times than those without AF termination. We intentionally did not include cumulative ablation time in our decision tree model because it may not reflect the efficacy of ablation as no contact force measurements were available at the time of the study. In contrast, the reduction of the LAA DF at the early steps of ablation may reflect the adequate elimination of critical AF drivers (Lemola et al., 2006; Honarbakhsh et al., 2018). Finally, we acknowledge that the DFs estimated by the Fourier analysis may lack temporal stability due to the non-stationarity of EGM signals during AF (Salinet et al., 2014). To address this potential limitation, the DFs were first computed on 10-s epochs and then averaged over multiple epochs, which has been shown to improve the DF reproducibility (Ng and Goldberger, 2007).

## Conclusion

This study showed that high surface and intracardiac DF values, and a negative LAA-to-RAA DF gradient before ablation are associated with the procedural failure to terminate AF and high recurrence rates at follow-up. Patients who achieved both a significant increase in LAA organization within 20 min of ablation following PVI and a low baseline RAA DF seem to benefit most from additional non-PV substrate modifications.

## Data Availability Statement

The original contributions presented in the study are included in the article/Supplementary Material, further inquiries can be directed to the corresponding author.

## Ethics Statement

The studies involving human participants were reviewed and approved by Human Research Ethics Committee of the Lausanne University Hospital. The patients/participants provided their written informed consent to participate in this study.

## Author Contributions

AP, J-MV, EP, and AL conceived and designed the study, analyzed the results, and drafted the manuscript. AP, AB, and AL performed the data analysis, statistics, and off-line database construction. EP and AL supervised the work and contributed to the final manuscript. AM, PP, ML, CH, C-IP, LR, MK, FS, SK, CS, and EP contributed to data collection and/or critical revision of the manuscript. All authors contributed to the article and approved the submitted version.

## Funding

This study was supported by Grant 18265.1 PFLS-LS from the Commission for Technology and Innovation (CTI), Switzerland and Grants SNF 205321 - 129876/1 and 146811/1 from the Swiss National Science Foundation.

## Conflict of Interest

The authors declare that the research was conducted in the absence of any commercial or financial relationships that could be construed as a potential conflict of interest.

## Publisher's Note

All claims expressed in this article are solely those of the authors and do not necessarily represent those of their affiliated organizations, or those of the publisher, the editors and the reviewers. Any product that may be evaluated in this article, or claim that may be made by its manufacturer, is not guaranteed or endorsed by the publisher.
