# Supplementary material for: Dynamics of Intraprocedural Dominant Frequency Identifies Ablation Outcome in Persistent Atrial Fibrillation
Source: Front Physiol. 2021 Oct 12;12:731917. doi: 10.3389/fphys.2021.731917 (PMC8546232; doi:10.3389/fphys.2021.731917)

SUPPLEMENTARY MATERIAL

1. Supplementary methods

1.1. Electrophysiological study

All patients had effective oral anticoagulation for ≥ one month. Antiarrhythmic drugs, with the exception of amiodarone and beta-blockers, were discontinued for ≥ five half-lives before the procedure performed under general anesthesia. Electroanatomical mapping and 3D reconstruction of the left atrium (LA) were performed with the CARTO® 3 system (Biosense Webster®). For each patient, the LA volume was computed after performing respiratory compensation and exclusion of the pulmonary veins (1).

1.2. Ablation strategy

Termination of persistent atrial fibrillation (peAF) was defined as the organization of AF into atrial tachycardia (AT) or into sinus rhythm (SR) during stepwise catheter ablation (step-CA). Supplementary Figure 1 describes the step-CA starting with pulmonary veins isolation (PVI), followed by CFAEs ablation based on a visual inspection (2), and LA linear ablation (roof and mitral isthmus). The study endpoint was reached when peAF was terminated into SR or AT during LA ablation. Non-terminated peAF were electrically cardioverted. At the end of step-CA, the effectiveness of PVI and bidirectional conduction block across the lines was confirmed with differential pacing and completed when needed. The ablation protocol was approved by the Human Research Ethics Committee of the Lausanne University Hospital and all patients provided written informed consent.

Repeated ablation procedures were performed for recurrent arrhythmia. The initial strategy included an assessment of PV reconnection for patients presenting with AF and ablation of micro and/or macroreentry for those presenting with ATs.

Clinical follow-up was performed at scheduled visits at 3, 6, 12, 18, and 24 months, then every year, and included echocardiographic evaluation, ECG and 48-h Holter recordings. Recurrence was defined as AF or AT lasting > 30 sec.

2. Supplementary figures and tables

Supplementary Figure 2 shows that all three patient subgroup displayed significand decrease in DF at the end of ablation.

Supplementary Figure 3 shows that only for the LT patients the extra-PV substrate ablation led to a durable abolition of the baseline positive LAA-to-RAA DF gradient.

Supplementary Table 1 shows that the baseline DF values are not predictive of long-term SR restoration by ablation.

Supplementary Table 2 shows that only the relative changes in LAA DF after 20 minutes of cumulative ablation within LA was associated with the long-term maintenance of SR. A decrease in LAA DF ≥6.61% predicted long-term maintenance of SR with 83% sensitivity, 74% specificity, 38% PPV and 96% NPV (AUC = 0.75, 95% CI 0.64-0.86, $p < 0.05$; Supplementary Table 2 and Supplementary Figure 4).

3. Supplementary references

1. Khan F, Banchs JE, Skibba JB, Grando-Ting J, Kelleman J, Singh H, et al. Determination of left atrium volume by fast anatomical mapping and intracardiac echocardiography. The contribution of respiratory gating. J Interv Card Electrophysiol. 2015 Mar;42(2):129–34.

2. Haïssaguerre M, Sanders P, Hocini M, Takahashi Y, Rotter M, Sacher F, et al. Catheter ablation of long-lasting persistent atrial fibrillation: critical structures for termination. J Cardiovasc Electrophysiol. 2005 Nov;16(11):1125–37.

Supplementary Table 1. Baseline ECG and EGM DFs as predictors of long-term sinus rhythm maintenance (LT_SR patients *vs* NLT + LT_Rec patients)

	Odds Ratio			ROC analysis					
	**OR**	**95% CI**	**p-value**	**AUC (95% CI)**	**Optimal cutoff**	**Se**	**Sp**	**PPV**	**NPV**
**RAA**	0.45	0.14 - 1.37	0.16	69% (0.49-0.88)	≤ 5.22 Hz	50%	87%	50%	87%
**LAA**	0.51	0.15 - 1.68	0.27	66% (0.47-0.85)	≤ 5.63 Hz	63%	71%	36%	88%
**CS**	0.72	0.22-2.29	0.57	56% (0.43-0.69)	≤ 5.58 Hz	75%	55%	30%	85%
**V_1**	0.51	0.15 - 1.64	0.26	64% (0.44-0.83)	≤ 5.74 Hz	75%	58%	32%	90%
**V_{6b}**	0.89	0.27 - 2.97	0.86	53% (0.36-0.71)	≤ 6.35 Hz	100%	13%	23%	100%
**LAA-to-RAA DF gradient**	1.95	0.90 - 1.23	0.48	58% (0.34-0.82)	≥ 0.35 Hz	50%	81%	40%	86%

AUC, area under the curve; CI, confidence interval; CS, coronary sinus; ECG, electrocardiogram; EGM, intracardiac electrogram; DF dominant frequency; LAA left atrial appendage; LT_Rec/LT_SR, left-terminated with/without recurrence at follow-up; NLT, not left-terminated; NPV, negative predictive value; OR, odds ratio; RAA right atrial appendage; ROC, receiver operating characteristic; PPV, positive predictive value; Se, sensitivity; Sp, specificity.

Supplementary Table 2. Prediction of long-term sinus rhythm maintenance using relative changes in DF after 20 min of cumulative left atrial CFAEs and linear ablation (roof and mitral isthmus).

	AUC (95% CI)	p-value	Decrease in DF	Se	Sp	PPV	NPV
**RAA**	65% (0.48-0.82)	0.09	≥ 2.45%	100%	45%	26%	100%
**LAA**	75% (0.64-0.86)	0.03	≥ 6.61%	83%	74%	38%	96%
**CS**	63% (0.50-0.70)	0.11	≥ 12.60 %	50%	90%	50%	90%
**V_1**	52% (0.32-0.71)	0.87	≥ 3.24%	83%	45%	23%	93%
**V_{6b}**	64% (0.43-0.85)	0.2	≥ 1.61%	100%	32%	22%	100%

Abbreviations as in previous tables.

Supplementary Figure 1. Stepwise radiofrequency catheter ablation protocol starting with pulmonary veins isolation (PVI), followed by left atrial CFAEs ablation and linear ablation of the roof and mitral isthmus. If atrial fibrillation (AF) was non terminated, a cardioversion was performed. Termination was achieved when the AF was converted into sinus rhythm (SR) or atrial tachycardia (AT).

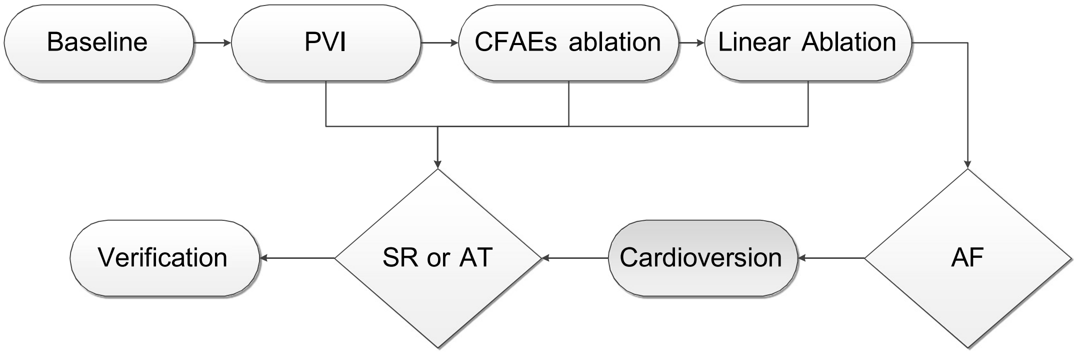

Supplementary Figure 2. Boxplots of relative changes in DF at the end of ablation (the last 3 min before AF termination or cardioversion) compared with the baseline DF values. DF was measured within the RAA, LAA, CS and on the ECG leads V_1 and V_{6b}. Abbreviations as in previous tables and figures.

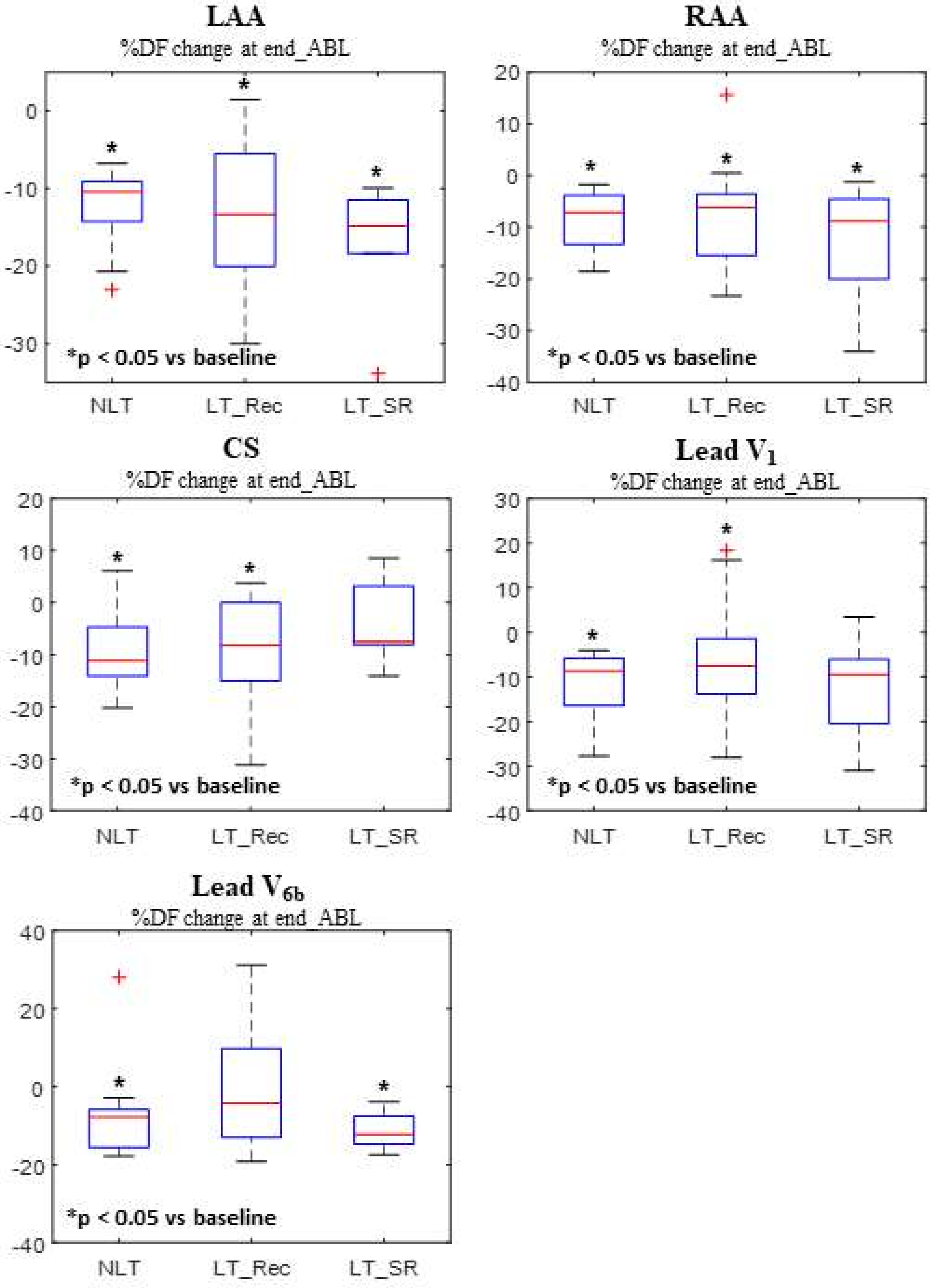

Supplementary Figure 3. LAA-to-RAA DF gradient at baseline (BL), at the end of PVI (end_PVI), during the first 10, 20, and 30 min of cumulative ablation following PVI, and the last 3 min of ablation (end_ABL). Abbreviations as in previous tables and figures.

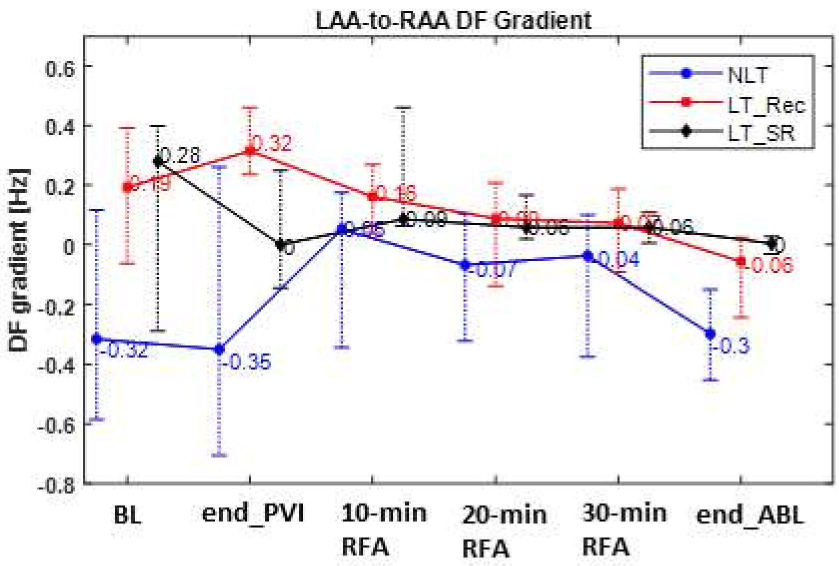

Supplementary Figure 4. Predictive performance of changes in LAA DF for long-term maintenance of sinus rhythm. (A) ROC curve analysis of relative changes in DF after 20 min of cumulative ablation following PVI. (B) Kaplan-Meier curves for freedom from AF recurrence after a single stepwise catheter ablation. Abbreviations as in previous figures and tables.

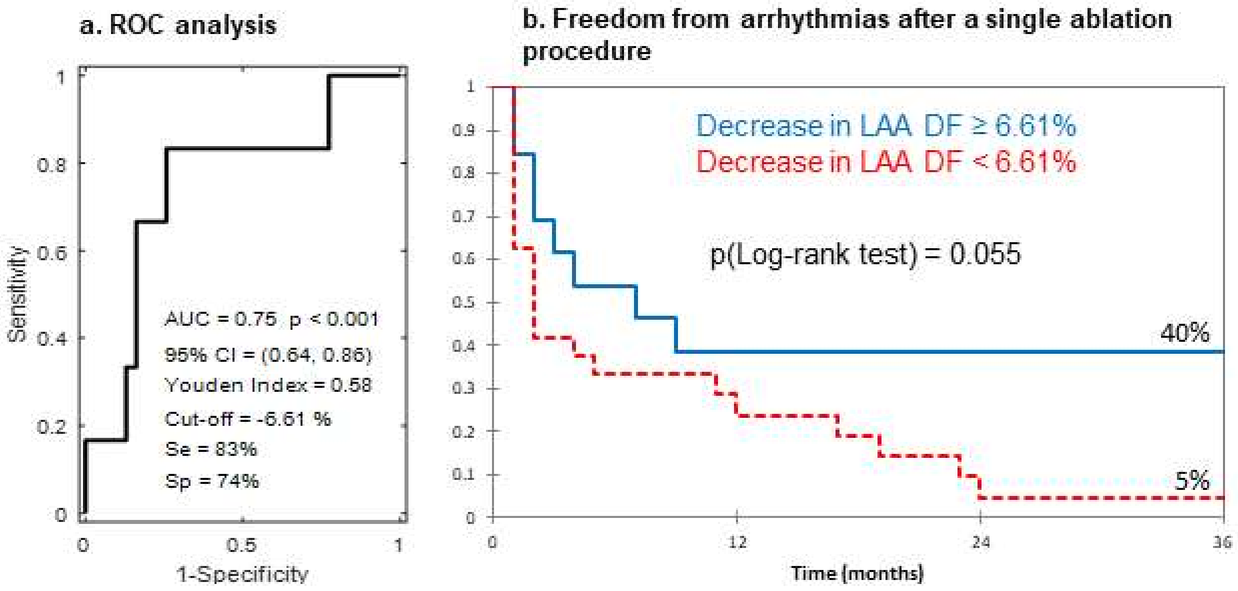

Supplement: Supplementary file 1 [file Data_Sheet_1.pdf]
